# Nanoformulations for Delivery of Pentacyclic Triterpenoids in Anticancer Therapies

**DOI:** 10.3390/molecules26061764

**Published:** 2021-03-21

**Authors:** Anna Kaps, Paweł Gwiazdoń, Ewa Chodurek

**Affiliations:** Department of Biopharmacy, Faculty of Pharmaceutical Sciences in Sosnowiec, Medical University of Silesia, Katowice, Poland, 8 Jedności Str., 41-208 Sosnowiec, Poland; pgwiazdon@wp.pl (P.G.); echodurek@sum.edu.pl (E.C.)

**Keywords:** pentacyclic triterpenoids, drug delivery systems, nanoformulations, nanocarriers, anticancer activity, civilization diseases

## Abstract

The search for safe and effective anticancer therapies is one of the major challenges of the 21st century. The ineffective treatment of cancers, classified as civilization diseases, contributes to a decreased quality of life, health loss, and premature mortality in oncological patients. Many natural phytochemicals have anticancer potential. Pentacyclic triterpenoids, characterized by six- and five-membered ring structures, are one of the largest class of natural metabolites sourced from the plant kingdom. Among the known natural triterpenoids, we can distinguish lupane-, oleanane-, and ursane-types. Pentacyclic triterpenoids are known to have many biological activities, e.g., anti-inflammatory, antibacterial, hepatoprotective, immunomodulatory, antioxidant, and anticancer properties. Unfortunately, they are also characterized by poor water solubility and, hence, low bioavailability. These pharmacological properties may be improved by both introducing some modifications to their native structures and developing novel delivery systems based on the latest nanotechnological achievements. The development of nanocarrier-delivery systems is aimed at increasing the transport capacity of bioactive compounds by enhancing their solubility, bioavailability, stability in vivo and ensuring tumor-targeting while their toxicity and risk of side effects are significantly reduced. Nanocarriers may vary in sizes, constituents, shapes, and surface properties, all of which affect the ultimate efficacy and safety of a given anticancer therapy, as presented in this review. The presented results demonstrate the high antitumor potential of systems for delivery of pentacyclic triterpenoids.

## 1. Introduction

Natural substances have been used in medicine for ages [1]. They may be useful in many civilization disorders like diabetes, obesity, and cardiovascular and neoplastic diseases [2,3]. Cancers are often characterized by an unknown etiology, high genetic instability, high histological heterogeneity, lack of specific biomarkers, and high local aggressiveness or spreading, all of which are usually challenging for modern medicine [4]. Despite the development in pharmacological sciences and the discovery of novel drugs for specific types of cancer, there are many factors that limit the possibility of their use. Toxicity to normal cells, the development of drug resistance, or a too short circulation in the body all ultimately add to the conclusion that cancer is one of the most common causes of death worldwide [5].

The strategies of anticancer therapies are most often based on the elimination of cancer cells by inhibiting their proliferation and on inducing the apoptosis process. It is extremely important that these properties should be limited to cancer without affecting normal cells. In recent years, there has been a growing interest in plant-sourced compounds that possess desired biological and pharmacological activities [6]. Pentacyclic triterpenoids, classified as phytochemicals, are secondary plant metabolites. They are most commonly found in the peel of fruits, leaves, and the bark of plants (e.g., the peel of apples, leaves of eucalyptus, and birch bark). Their physiological role is to protect against the harmful effects of microorganisms and insects. Plants with a particularly high content of pentacyclic triterpenoids could be used in the medical treatment called ‘phytotherapy.’ The pentacyclic triterpenoids can be classified into three main groups: lupane (betulin, betulinic acid, and lupeol), oleanane (oleanolic acid, maslinic acid, erythrodiol, and β-amyrin) and ursane (ursolic acid, uvaol, and α-amyrin) [7]. Their chemical structures consist of five- and/or six-membered carbon rings [8]. The range of biological properties of triterpenoids includes anticancer, antiangiogenic, anti-inflammatory, antiviral, antioxidant, antidiabetic, antihyperlipidemic, antibacterial, hepatoprotective, and cardioprotective activities, among others. Therefore, most triterpenoids are highly biologically active with a low toxicity that indicates the possibility to use them as an alternative to traditional chemotherapeutics [9,10].

## 2. Biological Activities of Pentacyclic Triterpenoids

### 2.1. Lupane-Type Triterpenoids

**Figure 1:**
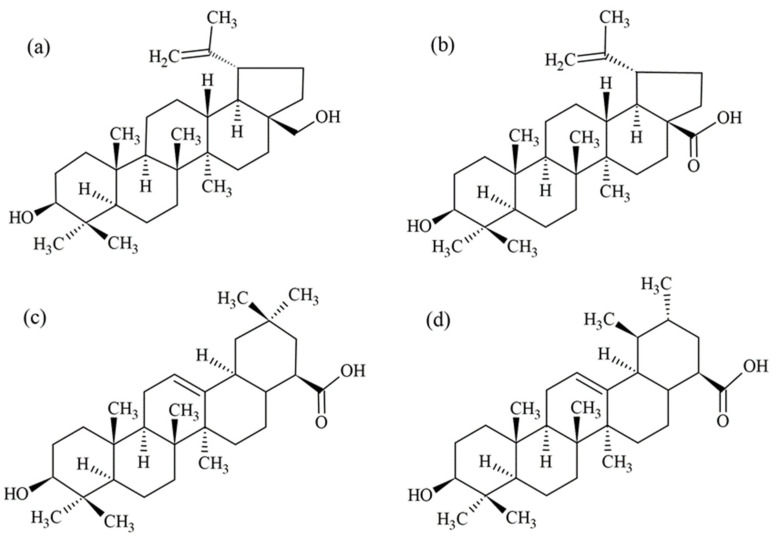
Chemical structures of betulin (**a**), betulinic acid (**b**), oleanolic acid (**c**), and ursolic acid (**d**).

Betulin (BT; lup-20(29)-ene-3β,28-diol; Figure 1a) also known as a betulin alcohol or betulinol, is one of the most studied and best characterized pentacyclic lupane-type triterpenoids. The highest concentration of BT (up to 80%) is found in birch bark extracts [11]. BT has two hydroxyl, at C3 and C28, and one isopropenyl, at C19, functional groups. Its extensive chemical structure is a very good substrate for numerous modifications. This enables the synthesis of appropriate derivatives with the desired characteristics [12].

**Figure 2:**
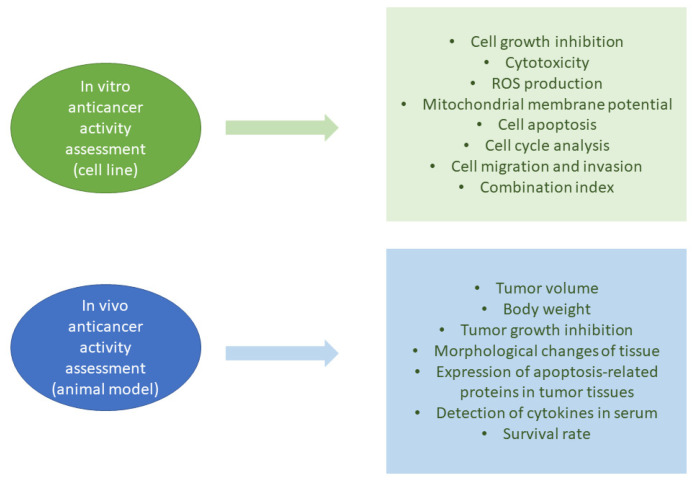
Markers for anticancer activity assessment. ROS: reactive oxygen species.

The most common betulin derivative is betulinic acid (BA; 3β-hydroxy-lup-20(29)-en-28-oic acid; Figure 1b). Among the lupane-type triterpenoids, BA is the most biologically active [11]. Unlike normal cells, BA impairs the antioxidant defense system, produces reactive oxygen species (ROS), and increases cytotoxicity in cancer cells. It can induce the cell cycle arrest and apoptosis of neoplastic cells. These properties can be used as markers for anticancer activity assessment (Figure 2).

BA regulates the expression of genes related to the apoptotic process and increases the translocation of proapoptotic Bcl-2-like protein 4 (Bax) and Bcl-2-antagonist/killer (Bak) protein into the mitochondria. The depolarization of the mitochondrial membrane increases the permeability and release of cytochrome c, Smac protein and apoptosis-inducing factor (AIF) into the cytosol, which, in turn, cleaves and activates the effector caspase-3 that is involved into the execution of apoptosis and cell death. Alternatively, BA can induce apoptosis in a CD95- and p53-independent and NFκB (nuclear factor-κ light chain enhancer of activated B cells)-dependent manner [13,14]. To date, the anticancer activity of lupane-type triterpenoids has been confirmed in leukemias; melanomas; neuroblastomas; medulloblastomas; glioblastomas; and ovarian, breast, prostate, colon, kidney, and hepatocellular carcinomas, among others [14,15].

### 2.2. Oleanane-Type Triterpenoids

A representative of the large oleanane group can be the oleanolic acid (OA; 3β-hydroxyolean-12-en-28-oic acid; Figure 1c) [16]. The most common source of OA in the human diet is the olive (*Olea europaea* L.) [7]. The chemical structure of OA contains the following functional groups: a hydroxyl group at C3 and a carboxyl group at C28 with an alkene moiety between C12 and C13 [17]. Its anticancer effect is based on the activation of AMPK (AMP-activated protein kinase), the suppression of complex crosstalk PI3K-Akt-mTOR (phosphoinositol 3 kinase-Akt-mammalian target of rapamycin)-NF-κB pathway, the upregulation of p53, and the activation of the apoptosis pathway. It can induce both extrinsic and intrinsic apoptosis pathways in numerous cancer cells derived from acute myeloid leukemia, liver, prostate, bladder, colorectal, and pancreatic cancer. Additionally, OA can affect cancer initiation, progression, and metastasis [18]. The co-administration of OA with sorafenib was found to increase the anticancer effect in hepatocellular carcinoma by ROS level enhancement [19]. Increased levels of ROS can induce not only apoptotic but also autophagic cell death [20]. The antiangiogenic activity of OA is based on the suppression of signal transducer and activator of transcription 3 (STAT3) and sonic hedgehog (SHH) signaling pathway activation, as well as the downregulation of proangiogenic vascular endothelial growth factor (VEGF) and basic fibroblast growth factor (bFGF). Moreover, OA is capable of exerting cytotoxic and proapoptotic effects in multi-drug resistant (MDR) cells like erythroleukemic cells that overexpress the glycoprotein (P-gp). Multi-drug resistance is one of the major causes of the failure of anticancer therapies. OA can be use both as an anticancer compound and an adjuvant in cancers with a high expression of efflux transporters. In non-antitumor use, the benefits of OA administration in liver diseases of varied etiology have been proven [18].

### 2.3. Ursane-Type Triterpenoids

The most commonly used in pharmacy ursane-type compound is ursolic acid (UA; 3β-hydroxy-urs-12-en-28-oic acid; Figure 1d). It possesses the same functional groups as the OA. UA is predominately found in the peel of fruits and the leaves of herbs [21]. It is involved in the modulation of some growth factors, like epidermal growth factor (EGF) and hepatocyte growth factor (HGF); receptors like epidermal growth factor receptor (EGFR); cellular transcription factors like STAT3 and NF-κB; and enzymes like caspase-3, -8, and -9. UA also decreases the production of pro-inflammatory cytokines, including interleukins IL-1, IL-6, and IL-8. Some studies have also shown the downregulating effect of UA on the expression level of matrix metalloproteinases (MMP-2 and MMP-9) and cell adhesive molecules (P-selectin, intercellular adhesion molecule 1) involved in tumor invasion and metastasis. The observed effect on cell proliferation, apoptosis, angiogenesis, metastasis, and autophagy can lead to irreversible changes and the death of cancer cells [22]. It has been reported that UA may inhibit the growth of pancreatic, prostate, lung, liver, gastric, breast, ovarian, and bladder tumors [23].

## 3. Drug Delivery Systems

**Figure 3:**
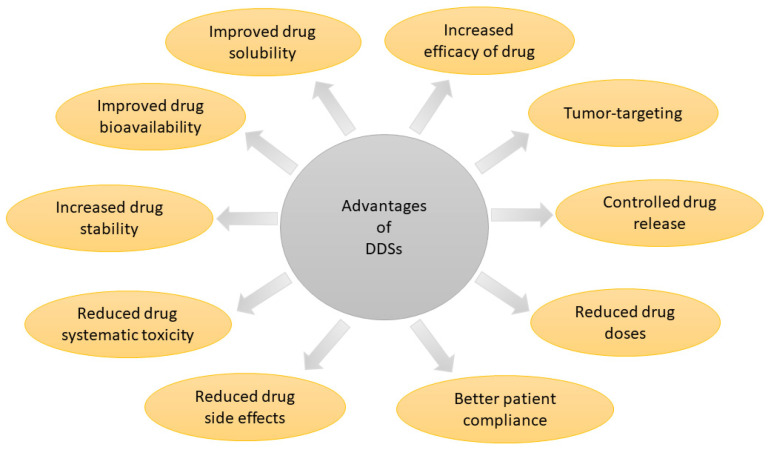
Advantages of drug delivery systems (DDSs).

Despite many beneficial properties, the use of pentacyclic triterpenoids in therapies is very limited. Their biological activities do not directly translate the results of experiments taken on cell lines or animal models into the clinical effectiveness of therapy in vivo. The bioavailability of pentacyclic triterpenoids is low, mainly due to their poor water solubility [24]. Moreover, they are grouped into the IV class of BCS (Biopharmaceutical Classification System), which is characterized by a low aqueous solubility, low intestinal permeability, and rapid elimination after oral administration. The poor solubility of compounds translates into a short half-life in blood circulation, low bioavailability, and the insufficient effectiveness of therapy [25,26]. On the other hand, after intravenous administration, the low-molecular-weight triterpenoids can undergo non-specific distribution in the body [27]. Pharmacological effects may be enhanced by the development of novel drug delivery systems (DDSs) based on nanocarriers. The use of nanocarriers for already available drugs or other biological agents can improve their therapeutic index and reduce toxic properties. This enables the improvement of pharmacokinetics, biodistribution of drugs, and effectiveness of therapy [28]. The overall benefits of DDSs are presented in Figure 3.

**Figure 4:**
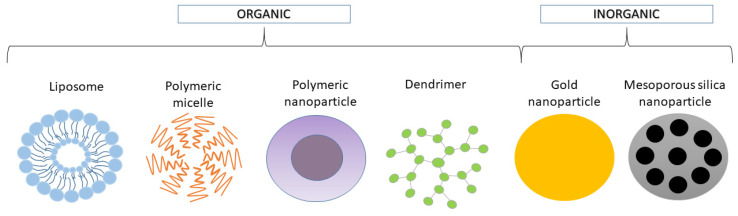
Frequently used types of organic and inorganic nanocarriers.

The nanocarriers used in DDSs predominantly act as pharmaceutical excipients and, less often, as active pharmaceutical ingredients (APIs). Pharmaceutical excipients can affect the solubility, transport, stability, permeability, bioavailability, and toxicity of the drug. When the carriers themselves have specific biological activities like antibacterial or anticancer, they can be classified as APIs [29]. The main goals of using nanocarriers in pharmaceuticals are increasing the stability of compounds, improving their transport to the site of action, and reducing the toxicity and risk of severe side effects in cases of compounds with a narrow therapeutic index. Based on their origin, nanocarriers can be made of organic and inorganic materials (Figure 4) [30].

### 3.1. Organic Nanocarriers

The first developed nano-size DDSs were lipid-based liposomes. They are spherical vesicles with hydrophilic interiors surrounded by one or more lipid bilayers formed as a result of the self-assembly of phospholipids. Their structure enables the encapsulation of hydrophilic drugs in a aqueous core and hydrophobic drugs into a membrane lipid bilayer [31]. For example, Caliskan et al. (2019) loaded liposomes with hydrophilic gemcitabine (GEM) and hydrophobic clofazimine, and they obtained a synergistic cytotoxic effect on osteosarcoma cells in vitro [32]. One of the greatest advantages of liposomes is the similarity of their membranes to native cell membranes, which facilitates cell–carrier interactions and content uptake. In case of multilayer liposomes, the compartmentalization and sequential release of the loaded compounds can be achieved [33]. Typically, nanocarriers can be recognized and eliminated by the reticuloendothelial system (RES). The RES uses phagocytic cells capable of recognizing and eliminating the foreign elements from body fluids. Recognition can be effectively limited by for example PEGylation. PEGylation is the attachment of polyethylene glycol polymer chains (hydrophilic component) to the surface of nanocarrier that affected the elimination pathway of a drug. It reduces aggregation and opsonization by plasma proteins, and it increases the half-life of the PEGylated structures in the blood system. In addition to PEGylation, other modifications like lipidation, glycosylation, and fusion to albumin may extend the half-time of nanocarriers and their biocompatibility [34].

**Figure 5:**
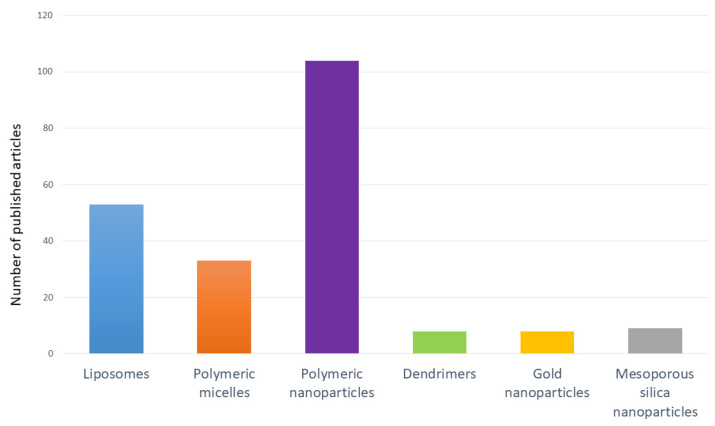
Number of published articles in last decade (2011–2021) about the use of selected DDSs with triterpenoids [37].

Polymers, both natural and synthetic, are the most commonly studied types of nanomaterials for manufacturing DDSs, including DDSs with triterpenoids (Figure 5). The properties of synthetic ones can be easily modified with the appropriate building blocks and synthesis method. Their most desirable features are biocompatibility, biodegradability, non-immunogenicity, and non-toxicity. Natural polymers, like chitosan or alginate, may also be useful in the manufacturing and coating of nanocarriers [35]. For example, the presence of chitosan on the carrier surface allows for the formation of pH-sensitive systems that can release loaded compounds into acidic environments [36]. Synthetic polyesters, like poly-ε-caprolactone (PCL), poly-L-lactide (PLA), and poly(lactic-co-glycolic acid) (PLGA) are biodegradable and biocompatible, so the risk of developing toxicity is significantly reduced [35]. The use of polymers, the structures of which can be precisely controlled, allows for the obtainment of molecules with specific and desired properties, like sensitivity to chemical (pH, temperature, and redox potential) or physical (light, ultrasounds, and magnetic field) stimuli. Polymeric materials can be used in the production of nanospheres, nanocapsules, micelles, polymerosomes, dendrimers, and many others. Depending on the type of DDS, the location of the drug in its structure is different. For example, in nanocapsules, the drug is placed in an inner, separate core, whereas in nanospheres, the therapeutic agent is dispersed in the polymer matrix [28].

Some polymers are able to self-assemble into nanoparticles. In the case of micelles and polymerosomes, the self-organization of amphiphilic molecules occurs. Hydrophobic agents can be entrapped in core of micelles, whereas hydrophilic compounds can be conjugated to the outer shell. In a non-aqueous solution, reverse micelles can be formed [38]. In turn, the inner cavity of a polymerosome is hydrophilic, so water-soluble compounds can be entrapped and transported in. The hydrophobic agents can be introduced into amphiphilic polymer bilayer [33]. The hydrophilic outer layer, in both micelles and polymerosomes, protects nanocarriers from non-specific uptake by immunological system components, stabilizes them in vivo, and can be easily modified by, e.g., PEGylation [33,38].

Hyperbranched dendrimers, made of polymers, are able to encapsulate some therapeutic molecules inside the dendrimer cavities or attach them to surface end groups of the nanocarrier. However, it should be mentioned that the charge of surface end groups can affect the biocompatibility, permeability, and toxicity of DDSs. The multitude of dendrimer surface groups allows for the achievement of the enhanced dendritic effect of loaded compound in comparison to free compound [29].

### 3.2. Inorganic Nanocarriers

Metallic nanoparticles (especially gold nanoparticles, AuNPs) and mesoporous silica nanoparticles (MSNs) are the most often used inorganic carriers [39]. AuNPs are characterized by a high drug-loading capacity, stability, and biocompatibility. A drug can be conjugated to the surface of AuNP via a gold–thiolate bond. The other possibility is non-covalent binding through hydrophobic or electrostatic interactions. AuNPs are capable of absorbing heat-generating near-infrared (NIR) light and can overheat neoplastic lesions via plasmonic photothermal therapy (PPTT). Despite the fact that gold is considered an inactive and chemically inert material, there have been some studies indicating that its toxicity and safety depends on the size, shape, and charge of the carrier, which requires further verification. PEGylation may be used to achieve the greater safety of AuNPs. Due to their properties, such as their high surface area to volume ratio, stability, multifunctionalisation, easy synthesis, and ability to photothermically convert, gold particles comprise one of the most interesting candidates in anticancer therapy [40,41].

The mesoporous silica nanoparticles used in DDSs are characterized by their high carrier porosity, which enables the introduction of biological agents into the interior, as well as high biocompatibility. Both hydrophilic and hydrophobic components can be placed in the pores of a carrier [42]. Introduced agents can be also connected by special linkers formed by functionalized silanol groups. Moreover, inside channels, high-molecular protein drugs or nucleic acid drugs can be encapsulated [30]. The use of MSNs is most often related to development of gatekeeper-based DDSs that release the therapeutic agents in sensitive-stimuli manner [43].

### 3.3. Passive and Active Targeting

**Figure 6:**
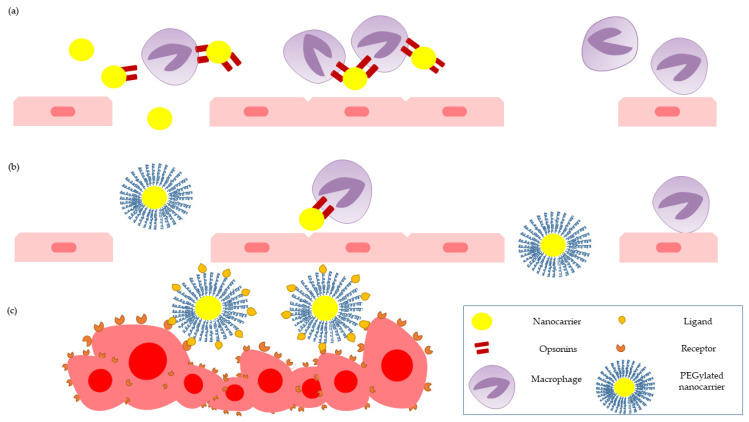
Nanocarrier-based drug delivery system for targeted and tumor-specific therapy: attaching opsonins to the “pure” surface of nanocarriers and reticuloendothelial system (RES) activation (**a**); passive targeting of PEGylated nanocarriers (**b**); active targeting of PEGylated and ligand-functionalized nanocarriers (**c**).

Conventional chemotherapy is based on low-molecular-weight (usually below 1000 Da) drugs. Due to their small size, chemotherapeutic agents, such as doxorubicin (DOX), gemcitabine, and cisplatin, have unfavorable pharmacokinetics and suboptimal bioavailability. This is evidenced by their short half-life and accumulation in non-target tissues. Their non-specific mechanism of action and the high volume of distribution can cause severe side effects such as neurotoxicity, myelosuppression, nausea, and vomiting in patients. Uncontrolled cancer cell proliferation and incomplete vascularization lead to the formation of tumors that can be easily permeable to nanocarriers (Figure 6). By increasing the diameter of systemically administered drugs, renal elimination can be reduced and the half-life of drug with accumulation at the site of action can be improved [44]. Additionally, the impairment of lymphatic drainage increases the retention, accumulation, and content release of nanodrugs. The enhanced permeability and retention (EPR) effect was first described in 1986 [45,46]. Due to numerous studies on the EPR phenomenon, its high heterogeneity has been observed. Heterogeneity in EPR-mediated tumor targeting can be attributed to changes in vessels (permeability, receptor expression, or maturation) or stroma (dense extracellular matrix, high cellularity, hypoxia, or interstitial fluid pressure). This translates into noticeable differences among individuals or tumor and metastases features in the same patient. Heterogeneity affects the effectiveness of drugs transported in nanocarriers. Interestingly, even variable drug accumulation in the surroundings of neoplastic lesions can provide better therapeutic efficacy than the use of standard forms of chemotherapeutic agents. The delivery of specific drugs to the site of action and the effectiveness of therapy can be improved by tumor-targeted drug transport. Tumor-targeting delivery systems are expected to increase drug concentration in cancerous tissues while limiting the delivery of drugs to normal tissues [44]. The improved concept of drug delivery involves the use of specific molecules called ligands. Surface-functionalized nanocarriers with ligands (bisphosphonates, aptamers, folic acid, and peptides, e.g., Arg-Gly-Asp domain, and hyaluronic acid) can interact with receptors on the surface of cells and pass inside the cell as a result of endocytosis [39]. It should be mentioned that PEGylation, due to the size of chains, can impair not only recognition by RES but also interactions between nanocarriers and target cells, and an additional ligand attachment may improve the active targeting and cellular uptake of nanocarriers. Taking advantage of these possibilities improves the overall effectiveness of anticancer therapy [47].

### 3.4. Challenges in Nanoformulations Design and Development

Despite the continuous development and undeniable overall benefits of the modern nanoformulations used in DDSs, there are still many unknowns and challenges that limit their widespread use. The current challenges for scientists in designing and manufacturing nanoformulations are related to (i) the properties of the used biomaterials, (ii) production limitations, (iii) loading capacity, (iv) stability, (v) toxicity, and (vi) biological barriers [27,30,48]. The properties of biomaterials, like charge of surface group or biodegradability, can affect their cytotoxicity, biocompatibility, and membrane permeability [29]. The manufacturing of nanoformulations should be optimized and easy to scale-up in order to achieve reproducible batches of the product without unfavorable production residues. Some of nanocarriers, like dendrimers, are also characterized by a very high production cost [49]. Increasing the loading capacity of carriers is necessary to achieve the appropriate concentration of the drug and the effectiveness of therapy without side effects [50]. The stability of nanoformulations enables the circulation of the drug-loaded nanocarriers and influences the release profile and dosing schedule of the administered drug. Though it is possible to extend the circulation of carriers in the patient’s body, it should be remembered that even PEGylation may contribute to the occurrence of unfavorable changes, i.e., difficult interactions with host cells and cellular uptake, excessive accumulation, and even the formation of anti-PEG antibodies [51]. Non-biodegradable PEGs and carriers, like gold nanoparticles or mesoporous silica nanoparticles, should be easily eliminated to reduce the risk of their toxic accumulation and undesirable effects in normal cells. The last but key aspect to be mentioned is the efficient transport and overcoming of biological barriers, e.g., the intestinal or blood–brain barriers, which so far constitute considerable challenges for research groups [52]. Along with overcoming the above-mentioned limitations, novel nanoformulations used in DDSs will be the basis of the future nanomedicine to provide more precise, controlled, and targeted therapy.

## 4. Nanoformulations with Pentacyclic Triterpenoids in Anticancer Therapy

The first developed DDSs were based on the use of liposomal nanoformulations [31]. Shu et al. (2019) evaluated the anticancer effect of BA-loaded liposomes that consisted of phosphatidylcholine, cholesterol, and mannosylerythritol lipid A (MEL-A) in a HepG2 cell line. Both BA and MEL-A were able to inhibit the progression of cell cycle in G1 phase after their introduction to liposomes. As a result, the percentage of cells in the S phase was significantly decreased. This inhibition was also observed as a noticeable decrease in the half maximal inhibitory concentration (IC_50_) value and was dose-dependent. Interestingly, liposomes modified with MEL-A but without embedded BA also contributed to a decline in IC_50_ that confirmed the antiproliferative properties of MEL-A-modified liposomes on human liver cancer cells. Moreover, MEL-A extended liposome stability and improved the interactions between cells and nanocarriers. By comparing the antiproliferative activity of BA, it can be seen that liposomes with embedded BA are more potent than free BA solutions. More detailed studies have found that BA-loaded liposomes induced apoptosis via the mitochondrial pathway, and the introduction of MEL-A in membrane bilayer enhanced disturbance of the mitochondrial membrane potential that could lead to intensive cell death. It can be concluded that additional modifications of liposome structures could improve compound delivery and the effectiveness of anticancer therapy [53]. Gao et al. (2012) encapsulated OA into liposomes with a protective PEG coating. The administration of the above-described nanoformulation to HeLa cells resulted in an increased cytotoxic effect on cancer cells. The in vitro antitumor activity was the highest in PEGylated liposomes in comparison to non-PEGylated nanocarriers or pure OA [54].

Wang et al. (2017) prepared OA-loaded liposomes modified by octreotide (Oct). Oct is a cyclic octapeptide with properties similar to somatostatin. The research results proved that the DDS with Oct had a greater antiproliferative activity and cellular uptake than the DDS without Oct in somatostatin receptor-positive A549 cells [55]. Sarfraz et al. (2017) investigated the co-loading of OA and DOX into the liposomal formulation in HepG2 cells. The key advantage of this system was the attenuation of the toxic effect of DOX on cardiomyocytes, while the synergistic anticancer effect of the DDS was noticeable at the same time [56]. De Araujo Lopes et al. (2013) synthesized PEGylated UA-loaded liposomes that consisted of cholesteryl hemisuccinate, dioleoylphosphatidylethanolamine, and distearoylphosphatidylethanolamine. These chemical components were involved in strong interactions between UA and lipid bilayer that led the liposomes remaining stable for up to 60 days [57]. In turn, Zhao et al. (2015) manufactured PEGylated UA-loaded liposomes that consisted of soya lecithin and cholesterol, which prolonged the release time and only 53.6% of total UA content was released within three days. The cumulative rate was 100% for free UA and 68.2% for non-PEGylated liposomes. In comparison to free UA, an increased antiproliferative effect in EC-304 cells, after 24 h of incubation with both non-PEGylated and PEGylated liposomes, was also observed. Results proved that the surface of liposomes can be functionalized to improve their stability and drug release profile [58]. Wang et al. (2017) showed that a chitosan coating on a liposomal surface could change the release profile of UA depending on the pH value. As the pH decreased from 7.4 to 5.5, the degree of release of the compound significantly increased from 35.7% to 100% within three days. Additionally, the accumulation of UA-loaded liposomes modified by chitosan in a cancer environment was found to be significantly greater compared to free UA [59].

Many research results have indicated the possibility of using polymeric materials in the development of DDSs for triterpenoid delivery. Polymeric DDSs have numerous advantages like easy functionalization, efficient drug-loading capacity, biocompatibility, a lack of immunogenicity, and diversity in manufactured structures. Zhou et al. (2019) developed UA-loaded polymer micelles (PMs) to improve clinical application of UA predominantly limited by a poor solubility, short half-time, and non-specific distribution in vivo. The prepared PMs consisted of a hydrophilic outer corona made from methoxy poly(ethylene glycol) and hydrophobic inner core formed by poly(L-lactide acid) with entrapped UA (UA mPEG-PLA PMs). The study was carried out on HepG2 cells and a normal L-02 cell line. The values of antiproliferative inhibitory parameter (IC_50_) after 24 and 48 h of the incubation of HepG2 cells with PMs were lower than with free UA at the same dosage. The same correlations were observed using a scratch-healing experiment that is useful in cell migration testing. The results of anti-hepatocarcinoma activity assessment in H22 xenograft mice were comparable with previous results, and PMs were stronger inhibitors of tumor growth and had a better impact on survival times of mice than free UA. Though a similar inhibitory effect was seen after the administration of 5-flurouracil (5-FU), the survival time was worse. The obtained results proved the hypothesis that UA-PMs were able to improve the triterpenoid properties and enhance antitumor effects on hepatocellular carcinoma with no effect on normal liver cells [60]. Zhang et al. (2013) observed that UA-loaded mPEG-PCL NPs showed a better inhibitory activity (IC_50_) in SGC7901 cells than free UA solutions. The high antitumor efficacy of NPs was achieved by the cyclooxygenase 2 (COX-2) suppression, overexpressed in gastric cancer, and caspase-3 activation [61]. Later research results of Zhang et al. (2015) proved that the incubation of hepatocellular carcinoma with manufactured UA-loaded poly(N-vinylpyrrolidone)-block-poly(ε-caprolactone) NPs (PVP-b-PCL NPs) also declined the IC_50_ values in comparison to free UA. The half-life of PVP-b-PCL NPs was more extended than that of mPEG-PCL NPs [62].

Polymers such as PLA and PLGA can also be used to produce UA-loaded NPs. They are characterized by an initial burst release (30%) during the first 15 min (PLGA NPs) or 8 h (PLA NPs), respectively. The further sustained release of UA could last from five days (PLA NPs) to 15 days (PLGA NPs) [63,64]. Wang et al. (2017) showed that Au-coated UA-loaded PLGA NPs could inhibit the progression of the cell cycle in cervical cancer cells (CaSki, HeLa, C4-1, and SiHa). The inhibition of cell migration and invasion with the simultaneous induction of the apoptosis pathway were also observed. No cytotoxicity in the 293T and L-02 cell lines was detected [65]. Silva et al. (2019) studied the anticancer properties of natural (NM) and synthetic (SM) mixtures of OA and UA using HepG2, Caco-2, and Y-79 cell lines. NM-OA/UA was extracted from *Plumeria obtusa* leaves, and SM-OA/UA was prepared with commercially available acids. Both mixtures were loaded into PLGA NPs. The obtained results in the HepG2 and Caco-2 cells indicated that the NPs reduced the toxicity of the loaded mixtures. In comparison to pure mixtures, NM, and SM, the NPs did not induce significant changes of cell viability. Moreover, cell incubation with pure SM resulted in a more decreased cell viability than with pure NM. The strong cytotoxic effect of 24 and 48 h of incubation with the tested mixtures was detected in Y-79 cells. After 48 h, the cell viability values reached 18.84% (NM-OA/UA), 12.24% (SM-OA/UA), 28.97% (NM-OA/UA NPs), and 21.01% (SM-OA/UA NPs), and they confirmed the possible use of these nanoformulations as prospective anticancer agents in retinoblastoma. The lower cytotoxicity of the OA/UA mixtures loaded in NPs compared to the pure mixtures indicated that they could be used in oral delivery systems to reduce potential intestinal toxicity in the case of incorrect administration [66]. However, it should be remembered that not all polymers are capable of forming effective triterpenoid-loaded NPs with anticancer properties. Oprean et al. (2016) found that the encapsulation of OA or UA in polyurethane nanoparticles (PU NPs) did not have a significant effect on the antitumor activity in breast cancer cells (MCF-7 and MDA-MB-231 cell lines) in contrast to pure compounds [67].

The PEGylation of the surface of nanocarriers and the co-encapsulation of chemotherapeutic agents can be useful tools in achieving optimal benefits from the produced DDSs. Saneja et al. (2019) assessed the possibility of manufacturing PEGylated PLGA NPs with hydrophobic BA and hydrophilic GEM (GEM-BA mPEG-PLGA NPs). GEM (2′,2′-difluorodeoxycytidine) is a nucleoside analogue commonly used as a chemotherapeutic agent in solid tumors. Unfortunately, it is characterized by a short half-time in the bloodstream and requires the administration of higher doses that can lead to the development of severe side effects such as myelosuppression, nephrotoxicity, and drug resistance [68]. In earlier studies, Pandita et al. (2014) confirmed the synergistic effect of GEM and BA solutions on MIAPaCa-2 and PANC-1 cancer cells. The development of GEM-BA-loaded mPEG-PLGA NPs affected antitumor efficacy and resulted in a synergistic effect in PANC-1 cells. The IC_50_ values were significantly decreased in comparison to GEM-loaded mPEG-PLGA NPs. The improvement in the effectiveness of therapy could have been a result of, among others, enhanced ROS production and the induction of apoptosis in the treated cells. The co-encapsulation led to an extension of the half-time of the used compounds. At the end of experiment, the volume of tumor was significantly reduced [69].

Targeted DDSs can improve the effectiveness of drug delivery to the site of action. This can be achieved by a ligand, e.g., lactoferrin (Lf), conjugating to the carrier surface. Lf is an iron-binding glycoprotein, belonging to the transferrin family, that is involved in iron metabolism, which is crucial in many life processes. Receptors for Lf are often overexpressed on the surface of metabolically active cancer cells and lead to an increased endocytosis of the compounds transported in Lf-conjugated carriers [70]. Moreover, Lf-functionalization facilitates the crossing of the blood–brain barrier (BBB). Halder et al. (2020) investigated the interactions between Lf and transferrin receptors on the surface of triple negative breast cancer (TNBC) cell line—MDA-MB-231. Since they were from a metastatic cancer cell line, human larynx epidermoid carcinoma Hep-2 cells were used. The Lf-conjugated, BA-loaded PLGA NPs had a strong impact on the IC_50_ values and levels of subG1 cell population in comparison to free BA. The results showed that Lf-modified BA PLGA NPs had a potent antiproliferative and cytotoxic effect on both cancer cell lines [71]. The lactoferrin functionalization of NPs was also used in OA nanoformulations. Xia et al. (2017) developed Lf-OA-loaded NPs that could improve the in vivo oral absorption and bioavailability of poorly water-soluble compounds. The bioavailability in male Sprague Dawley rats was nearly 3.4-times greater than in case of the administration of free OA [25].

Another possible functionalization is the attachment of the folate (FA) to the nanocarrier surface, which enables receptor-mediated endocytosis via the folate receptor (FR). Gao et al. (2015) synthesized dendrimeric prodrugs based on polyamidoamine (PAMAM) conjugated with UA and FA (FA-G3/G5-UA). The release of UA and ester bond hydrolysis were controlled by the pH value. The occurrence of FA on the surface enhanced the cellular uptake of dendritic nanoformulations (differences between FR-positive and FR-negative cell lines). Moreover, FA-modified dendrimeric prodrugs led to a greater increase in cytotoxicity in FR-positive HeLa cells than non-FA-modified PAMAM dendrimers [72]. Jin et al. (2016) modified UA-loaded NPs with not only chitosan but also folate residues (FA-CH-UA NPs), which had an impact on drug releasing profile and enabled the endocytosis of the biological agent via folate receptors on the surface of MCF-7 cells [73]. Shen et al. (2018) manufactured self-assembled PAMAM dendrimers with UA and lactobionic acid (UA2-G0-LA). The cytotoxicity of dendrimers against SMMC7721 cancer cells was enhanced compared to a control. Dendrimers also suppressed metastasis through changes in the migration and adhesion of investigated cells. An in vivo study on H22 mice model confirmed the prolongation of plasma half-time and the inhibition of tumor growth [74]. Liu et al. (2018) developed self-assembling drug conjugates consisting of pectin, 8-ArmPEG, UA, and hydroxycamptothecin (Pec-8PUH NPs). This DDS ensured an increased stability, prolonged clearance, and half-life of the drug in comparison to free UA. After the administration of Pec-8PUH NPs, the synergistic effect of UA and 10-hydroxycamptothecin (HCPT) in 4T1 cells was observed. The survival rate in 4T1 tumor-bearing mice was enhanced in comparison to free compounds [75]. In their previous studies, Liu et al. (2017) used carboxymethylcellulose instead of Pec-8-ArmPEG. As a result, an extended drug retention time, inhibition of tumor growth, and improvement in anticancer activity and survival rate were obtained [76].

Wang et al. (2020) prepared amphiphilic polyprodrug poly(oligo(ethylene glycol) methyl ether methacrylate)-b-poly(oleanolic acid methacrylate) (POEGMA-b-POAMA/HCPT NPs) that consisted of hydrophilic POEGMA and hydrophobic OA prodrug monomers. Additionally, NPs were able to entrap the 10-hydroxycamptothecin in their core. In vitro studies were performed on MCF-7 and 4T1 cell lines and indicated that the OA and HCPT release rates were quite similar, with a sustained release for up to 132 h in the acidic environment. A significant cytotoxic effect against 4T1 and MCF-7 cells was detectable. Furthermore, in vivo studies using a 4T1 xenograft tumor murine model showed that POEGMA-b-POAMA/HCPT NPs had a greater antitumor efficacy with minimal adverse effects in comparison to POEGMA-b-POAMA and free HCPT [77].

Mioc et al. (2018) investigated the properties of BT-conjugated AuNPs with or without thiolated PEG (PEG-SH) in A375 and B164A5 melanoma cell lines. The medium molecular weight PEG-SH molecules were used to achieve more stable and biocompatible nanostructures. A cytotoxic effect and the induction of apoptosis were detected in both cell lines and were dose- and time-dependent. The obtained results proved previous assumptions that BT-loaded AuNPs could improve the drug bioavailability and anticancer properties of triterpenoids. Interestingly, both PEGylated AuNPs with BT and non-PEGylated AuNPs without BT had no significant effect on cell viability [78].

Non-organic NPs can be useful in reversing the multidrug resistance of cancer cells. Li et al. (2020) determined the possible use of hybrid NPs with cisplatin and OA (HN/CDDP/OA) in the therapy of gastric cancer. The HNs were prepared from the membrane of MGC-803 cancer cells mixed with an aqueous solution of calcium carbonate (CC). The binding between calcium ions and the phosphates of the membrane led to the improved stability and targeting of the DDS [79]. Cisplatin is a commonly used chemotherapeutic agent involved in the formation of DNA adducts, which leads to DNA damage and apoptosis induction in cancer cells [80]. The investigated NPs were stable and biocompatible. The drug release profile, in the case of both compounds, was pH-dependent, which is a crucial aspect in anticancer strategy due to the acidic extracellular pH in cancer tissue. Tumor-specific drug targeting and accumulation with the intensification of apoptosis processes and the withdrawal of MDR cells in both the in vitro and in vivo conditions of experiment were observed [79].

DDSs based on MSNs have many advantages like a high surface area, a large pore volume, mechanical stability, biocompatibility, and easy surface functionalization. Li et al. (2017) proved that UA-loaded MSNs showed a more potent cytotoxic effect on HepG2 cells than free UA. The rate of release of UA was pH-dependent [81]. Jiang et al. (2017) developed UA-loaded MSNs that could be functionalized using chitosan and folate residues. Cell proliferation and invasion were decreased through the cell cycle arrest in the G0/G1 stage. The advantages of using such a DDS were confirmed in mice, where the administration of MSNs significantly inhibited tumor growth and lung metastasis [82]. Zhao et al. (2017) manufactured a system that was intended to provide the pH-dependent release of not only UA but also sorafenib. Chitosan and LA-modified UA-loaded MSNs improved cellular uptake and drug internalization in an asialoglycoprotein receptor (ASGPR) overexpressing SMMC7721 cancer cells. Moreover, in vivo MSNs were capable of inhibiting lung HCC metastasis, which could be useful strategy in patients with hepatocellular carcinoma [83].

Recently, novel DDSs based on the self-assembling properties of triterpenoid molecules were developed. Many chemotherapeutic agents must be administered in high concentrations, in multiple doses, or in combination systems to achieve optimal effectiveness in cancer therapy. NPs can protect drug molecules, extend their half-time in the bloodstream, sustain drug release, and improve tumor-targeting. A very advantageous strategy in anticancer therapy is the use of compounds that not only have the desired biological properties but are also capable of self-assembly to nanocarrier particles. This could reduce the risk of adverse drug events and increase the amount of drug delivered to the site of action. An increased frequency of breast cancer brain metastases (BCBMs) has been seen in patients with advanced breast cancer. Chemotherapy is often not effective in BCBM due to the limitations of the efficient transport of drug across the BBB and the necessity to use multidirectional combination therapy. In this case, DDSs may be the optimal therapeutical solution. Bao et al. (2020) discovered that OA was able to self-organize into spherical OA NPs. The supramolecular self-assembly formed through interactions between hydrogen bonds. The OA NPs not only had anticancer properties themselves but also were able to encapsulate an anticancer drug like paclitaxel (PTX). PTX is a commonly used chemotherapeutic compound in clinical practice. Unfortunately, its water solubility and bioavailability are limited. Both PTX and OA were found to affect proliferation by inhibiting cell cycle progression in the G2/M phase, the induction of autophagy, and apoptosis in MDA-MB-231 and MCF-7 cells. Interestingly, OA could inhibit the efflux transporters, including P-gps, involved in PTX elimination and then increase PTX intracellular concentration—hence its anticancer effect. The administration of PTX-loaded OA NPs resulted in a synergistic inhibitory effect on both cell lines. In vivo studies proved the effective penetration of tumor through leaky vessels, as well as a synergistic effect on a primary breast tumor and its more advanced feature, the BCBM [48].

In turn, Ou et al. (2020) assessed the possibility of using poly(ursolic acid) nanoparticles (PUA NPs) formed by the polycondensation of UA, in which PTX could also be encapsulated. Polycondensation was possible due to occurrence of hydroxyl and carboxyl groups in the UA native structure. The obtained results showed that PUA NPs could extend the circulation time and enhance the accumulation of nanocarriers in colorectal cancer (CRC) tissues. PUA NPs were characterized by a good biocompatibility with a strong cytotoxicity detected in neoplastic cells, which was probably the result of increased cellular uptake compared to free UA. In comparison to pure UA, the number of cells arrested in the G2/M phase and the delayed tumor progression after the administration of PUA NPs were also greater. Both in vitro (CT26 cells) and in vivo (CT26 tumor-bearing mice) studies have confirmed the antitumor activity of PTX-loaded PUA NPs and PUA NPs with no severe side effects [84].

Recent reports described the research of the team of Colombo et al. (2020) who developed the BA-based self-organized NPs with cabazitaxel, podophyllotoxin, or N-desacetyl thiocolchicine. Betulinic methyl ester could be used as either a self-assembly inducer or a structural unit conjugated via a linker (sebacic acid) with tubulin binders. Moreover, in the case of N-deacetyl thiocolchicine conjugation, a triazole-based linker was also used. The ovarian cancer cell line A2780 was incubated with either self-assembled NPs or conjugates. The highest cytotoxic effect was determined in cells treated with thiocolchicine-based conjugates, especially with a thiocolchicine–triazole conjugate. The largest difference in the cell growth inhibition value (GI_50_) was noticeable between the conjugates and the NPs of cabazitaxel. Possible explanations for this effect were either the partial hydrolysis of the ester linkage used for linker attachment or the slow disaggregation of NPs. The obtained results indicated the significant potential for clinical use of such tumor-targeting DDSs [85].

The collected information about the above-mentioned nanoformulations is presented in Table 1.

## 5. Conclusions

Despite the development of many therapeutic strategies, the therapy of neoplastic lesions is still ineffective. Until now, chemotherapy and surgery had been the most often used strategies, depending on the location and stage of the tumor. Effective anticancer therapy may be based on various mechanisms of action of the administrated drug, e.g., the inhibition of cell proliferation, ROS production, the induction of apoptosis, the inhibition of angiogenesis, and cell migration [9]. Pentacyclic triterpenoids are widely distributed and can be easily sourced from various plants. They comprise a group of compounds characterized by a wide range of biological properties useful in the treatment of many diseases, including neoplastic lesions. However, it should be remembered that there are significant limitations to their clinical use, especially their low water solubility, low bioavailability, and rapid metabolism of free compounds [12,86]. These properties may be changed by introducing chemical modifications or using optimized delivery systems based on nanocarriers or the self-assembly of molecules. It is extremely important to develop novel formulations of compounds that increase the bioavailability of phytochemicals grouped into the IV class of BCS.

The use of novel nanoformulations with pentacyclic triterpenoids improves their effectiveness in anticancer therapy. This review showed a multitude of solutions that could be adapted to the type of cancer and the expected results. In most of the presented studies, cell proliferation was decreased (Table 1). Some nanoformulations acted as potent cell cycle inhibitors in the G1 [53] or G2 [48,84] phases. Generally, nanoformulations with BA, OA, and UA have been found to have a greater antiproliferative and cytotoxic effect than free compounds. An exception was observed in the Silva et al. (2019) study, in which the toxicity of NM-OA/UA and SM-OA/UA-loaded NPs were reduced in HepG2 and Caco-2 cells and cell growth did not significantly change compared to pure mixtures. In turn, the same mixtures loaded in NPs had a strong anticancer effect on retinoblastoma Y-79 cells [66].

In all presented studies, no cytotoxic effect was observed in normal cell lines. This confirmed the selectivity of the used therapeutic strategies. A greater tumor-targeting was achieved through the functionalization of carriers by binding folate [72,73], lactoferrin [25,71], or lactobionic acid [74] residues. This could also improve the cellular uptake of nanoformulations. In turn, PEGylation was found to increase the stability of nanocarriers, extended their circulation time and reduced recognition by the RES [34]. This contributed to increasing the anticancer activity of the administered compounds. In the De Araujo Lopes et al. (2013) study, strong interactions between lipids and UA led to the development of nanoformulations that were stable for up to 60 days [57]. At this point, it should also be mentioned that not all polymer nanoparticles can increase the anticancer effect of triterpenoids. OA or UA-loaded polyurethane nanoparticles did not have an impact on antitumor activity in breast cancer cells in contrast to free compounds [67].

Scientists’ efforts are increasingly focused on developing precise and effective therapies for individuals. One of the future directions is the further development, design, and synthesis, the so-called “smart” nanoparticles. The term “smart” refers to changes in the properties of nanocarriers that lead to stimuli-responsive drug release from the carriers [87]. For example, the use of chitosan on the surface of nanocarriers leads to a rapid release of the drug in an environment with a lowered pH, which is characteristic of the cancer tissue microenvironment [59,73,83]. Additionally, the targeting of drug carriers to the appropriate regions or compartments in a patient’s body will enhance the drug efficiency and reduce the risk of side effects of therapy (e.g., above-mentioned functionalization via folate, lactoferrin, or lactobionic acid). Future therapies will not be based on the use of one drug. Thanks to the use of specific nanoformulations, it will be possible to develop combined therapies with several biological agents released in a changed environment. This will also reduce the risk of multi-drug resistance. The use of several substances is most often aimed at achieving a synergistic effect and a better therapeutic index with a simultaneous reduction in doses and toxicity, e.g., chemotherapeutic agents [27]. The presented research results confirmed the possibility of introducing two different anticancer compounds, e.g., UA and HCPT [75], BA and GEM [68], OA and PTX [48], and OA and DOX [56], into nanocarriers. This can also reduce the toxicity of chemotherapeutic agents such as DOX [56] and PTX [48,84]. Another interesting trend is the idea of creating multifunctional systems, so-called theragnostic, that combine diagnostic and therapeutic factors in one nanoformulation. Theragnostic nanoparticles should accumulate at the site of action, be able to assess the biochemical and morphological properties of damaged cells, release the drug, and be easily eliminated from the body without developing undesirable effects [88].

The presented research results indicated a high potential for the development of nanoformulations using inorganic carriers, especially gold ones, due to their stability, multifunctionalisation, and ability to be used in PPTT [41]. Improving drug delivery systems with pentacyclic triterpenoids will “shift” research from preclinical to clinical trials that will determine the potential for their accurate use in oncological practice.

The results of previous publications showed that the compound with the greatest anticancer potential of the pentacyclic triterpenoid group is ursolic acid. UA is capable of inhibiting of cell proliferation [81], invasion [60,65], and metastasis [74,83]. Due to the progress of nanomaterials, the assessment of the biological activity of precursor compounds for OA and UA, i.e., α- and β- amyrines, could also be considered. Earlier research results indicated the possibility of introducing amyrins into nano emulsions [89] or β-cyclodextrins [90]. Their possible anticancer activity should be evaluated in novel nanoformulations.

The use of self-assembling nanocarriers with anticancer activity is one of the most interesting and promising directions for further research. It could reduce the risk of adverse drug events and increase the amount of drug delivered to the site of action [48,84,85]. Further exploration at the levels of in vivo and multidisciplinary studies carried out over extended periods of time that assess the benefits and drawbacks of such systems is needed [28]. All these efforts will most likely contribute to improvement of the effectiveness of anticancer therapy and the quality of life of patients.

## Figures and Tables

**Table 1:** The most important information about nanoformulations with pentacyclic triterpenoids.

Pentacyclic Triterpenoid	DDSs	Cell Line	Animal Model	Effects	Reference
Betulin	Gold nanoparticles	A375, B164A5, 1BR3, and HaCaT	−	Increased cytotoxicity and induction of apoptosis.	[78]
Betulinic acid	Liposomes	HepG2	−	Inhibition of cell cycle, increased stability, and induction of apoptosis.	[53]
	Polymeric nanoparticles	PANC-1	Ehrlich Ascites Carcinoma in Swiss albino male mice	Decreased cell proliferation, enhanced ROS production, induction of apoptosis, and reduced tumor volume.	[68]
		MDA-MB-231 and HEp-2	−	Decreased cell proliferation and increased cytotoxicity.	[71]
	Self-assembled nanoparticles	A2780	−	Increased cytotoxicity and decreased cell proliferation.	[85]
	Polymer-drug conjugates	A2780	−	Increased cytotoxicity and decreased cell proliferation.	[85]
Oleanolic acid	Liposomes	HeLa	−	Increased cytotoxicity.	[54]
		A549	−	Increased cellular uptake and decreased cell proliferation.	[55]
		HepG2, HepG3B, H9C2, and L-02	HepG2 tumor-bearing female BALB/c mice and female Kunming mice	Increased anticancer activity and decreased doxorubicin (DOX) toxicity.	[56]
	Polymeric nanoparticles	MCF-7, T47D, MDA-MB-231, and MDA-MB-361	−	No significant effect on anticancer activity.	[67]
		−	Male Sprague Dawley rats	Improved oral absorption and bioavailability.	[25]
	Hybrid nanoparticles	MGC-803 and NIT3T3	MGC-803 tumor-bearing male BALB/c mice	Increased stability, biocompatibility and tumor-targeting, and induction of apoptosis.	[79]
	Self-assembled nanoparticles	4T1 and MCF-7	4T1 tumor-bearing female BALB/c mice	Sustained drug release and increased cytotoxicity.	[77]
		MDA-MB-231-WT, MDA-MB-231-BR, MCF-7, and NHA	MDA-MB-231-WT tumor-bearing female athymic NCr-nu/nu mice, and MDA-MB-231-BR tumor-bearing female athymic NCr-nu/nu mice	Inhibition of cell cycle, improved paclitaxel (PTX) bioavailability, induction of autophagy and apoptosis, and inhibition of efflux transporters.	[48]
Ursolic acid	Liposomes	MDA-MB-231 and LNCaP	−	Improved stability and decreased cell proliferation.	[57]
		EC-304	−	Sustained drug release, decreased cell proliferation, and increased stability.	[58]
		HeLa	U14 tumor-bearing female CD-1 mice	Increased tumor-targeting.	[59]
	Polymeric micelles	HepG2 and L-02	H22 tumor-bearing male Kunming mice	Decreased cell proliferation, decreased cell migration, and increased survival time.	[60]
	Polymeric nanoparticles	SGC7901	−	Decreased cell proliferation, decreased cyclooxygenase 2 (COX-2) expression and increased caspase-3 activity.	[61]
		H22	H22 tumor-bearing ICR mice	Decreased cell proliferation and increased stability.	[62]
		B16F10	−	Increased cytotoxicity and sustained drug release.	[63]
		B16F10	B16F10 tumor-bearing male BALB/c mice	Increased cytotoxicity, increased cellular uptake, and sustained drug release.	[64]
		CaSki, HeLa, C4-1, SiHa, 293T, and L-02	CaSki, HeLa, and SiHa tumor-bearing male athymic nude mice	Inhibition of cell cycle, cell migration, and invasion, as well as an induction of apoptosis.	[65]
		HepG2, Caco-2, and Y-79	−	Differentiated impact on cell proliferation and cytotoxicity.	[66]
		MCF-7, T47D, MDA-MB-231, and MDA-MB-361	−	No significant effect on anticancer activity.	[67]
		MCF-7 and Colo205	MCF-7 tumor-bearing female BALB/c mice	Increased tumor-targeting and cellular uptake.	[73]
	Dendrimers	HepG2 and HeLa	−	Increased tumor-targeting and cellular uptake.	[72]
		SMMC7721 and HeLa	H22 tumor-bearing Sprague Dawley rats and H22 tumor-bearing Kunming mice	Increased cytotoxicity and tumor-targeting, decreased migration, adhesion, metastasis, and tumor growth.	[74]
	Polymer-drug conjugates	4T1 and MCF-7	4T1 tumor-bearing female BALB/c mice	Increased stability and survival rate.	[75]
		4T1	4T1 tumor-bearing female BALB/c mice	Increased stability and survival rate, decreased tumor growth.	[76]
	Mesoporous silica nanoparticles	HepG2	−	Increased cytotoxicity.	[81]
		HeLa	H22 tumor-bearing nude mice	Decreased cell proliferation, invasion and metastasis, as well as the inhibition of the cell cycle.	[82]
		SMMC7721, HepG2, Huh-7, and HeLa	H22 tumor-bearing nude Kunming mice	Increased cellular uptake and decreased metastasis.	[83]
	Poly(ursolic acid) nanoparticles	CT26 and NIH 3T3	CT26 tumor-bearing male Sprague Dawley rats and CT26 tumor-bearing male BALB/c mice	Increased stability, accumulation in cancer tissues, cytotoxicity and cellular uptake, and inhibition of the cell cycle and tumor progression.	[84]
